# Different Traditional Herbal Medicines for the Treatment of Gastroesophageal Reflux Disease in Adults

**DOI:** 10.3389/fphar.2020.00884

**Published:** 2020-07-16

**Authors:** Yun-kai Dai, Yun-bo Wu, Hao Wen, Ru-liu Li, Wei-jing Chen, Chunzhi Tang, Liming Lu, Ling Hu

**Affiliations:** ^1^ Institute of Gastroenterology, Guangzhou University of Chinese Medicine, Guangzhou, China; ^2^ Science and Technology Innovation Center, Guangzhou University of Chinese Medicine, Guangzhou, China; ^3^ Medical College of Acu-Moxi and Rehabilitation, Guangzhou University of Chinese Medicine, Guangzhou, China

**Keywords:** traditional herbal medicines, gastroesophageal reflux disease, randomized controlled trials, network analysis, adults

## Abstract

**Background/Aims:**

Traditional Herbal Medicines (THM) have been being used for gastroesophageal reflux disease (GERD) for a long time, but clinical evidence is still scarce. We evaluated different THM prescriptions for GERD in adults.

**Methods:**

Data added to nine online databases from their inception to November 30, 2019, were systematically searched. All relevant randomized controlled trials (RCTs) were included and were combined with Bayesian network analysis. The Cochrane Collaboration’s risk of bias tool and GRADE profiler version 3.6 were respectively employed to evaluate the quality of evidence of outcomes.

**Results:**

Seventeen publications involving 1441 participants were retrieved. The results of our analysis suggested that Jianpi therapy+proton pump inhibitors (PPIs) and Ligan Hewei therapy respectively ranked first in overall clinical efficacy and efficacy under gastroscope; Ligan Hewei therapy+PPIs was the optimum intervention in the improvement of acid regurgitation and heartburn.

**Conclusion:**

This research indicates that Ligan Hewei therapy and Jianpi therapy, or these therapies separately combined with PPIs, should be recommended as appropriate complementary and alternative treatments based on the specific characteristics of GERD. However, additional well-designed RCTs with high methodological quality are still needed for future research.

## Introduction

Gastroesophageal reflux disease (GERD) is a common chronic disorder characterized by an imbalance of the barrier between the stomach and the esophagus, resulting in the regurgitation of gastric contents into the esophagus amd even the hypopharynx (DeVault and Castell, 2005). Based on the Montreal definition published in 2006, it is subclassified into non-erosive reflux disease (NERD), reflux esophagitis (RE), and Barrett esophagus (BE) (Vakil et al., 2006). Moreover, epidemiological investigation showed that this disease affected approximately 20%~30% of the population around the world and 7.8%~8.8% in East Asia (El-Serag et al., 2014). Without timely treatment, patients with the condition will suffer from numerous complications including esophageal stricture, ulceration, and even BE (Freston et al., 1995; Schwizer and Fried, 1997), thereby leading to huge psychological burden and poor work productivity (Wahlqvist et al., 2008; Nocon et al., 2009).

Currently, the first-line medical drug for GERD is proton pump inhibitors (PPIs). They are estimated to provide about a 56%~76% rate of relief of related symptoms and an 80%~85% recovery rate for esophageal lesions (Katz et al., 2006) as well as reducing the incidence of complications (Savarino et al., 2009). However, approximately 30% GERD sufferers, who had unsatisfactory responses to PPIs still remained symptomatic and had high risk of complications, including BE (Fass et al., 2005). Therefore, in order to seek other effective therapies and improve their quality of life, many patients put their attention on alternative medicine (Patrick, 2011).

The use of traditional Chinese medicine (TCM) has a long history and was first documented by the *Sheng Nong Classic of Materia Medica*. Currently, traditional Herbal Medicines (THM) are widely used in cardio-cerebrovascular, endocrine, gastrointestinal, neuropsychiatric, and respiratory disorders (Dai et al., 2018; Jiang et al., 2019; Kong et al., 2019; Qin et al., 2019; Gao et al., 2020; Liu et al., 2020; Zhang et al., 2020). Several studies have evaluated the efficacy and safety of THM in treating GERD (Ling et al., 2015; Dai et al., 2017; Shih et al., 2019). However, these findings were obtained from pairwise comparisons between a prescription and conventional Western medicine(s). No comparison within different THM was conducted in the treatment of GERD. Consequently, to obtain up-to-date information regarding the effectiveness of different prescriptions in treating this disorder, a Bayesian network analysis that integrated direct with indirect evidence for multiple intervention comparisons was performed in this study.

## Methods

This study was performed based on the Preferred Reporting Items for Systematic Review and Meta-Analysis (PRISRMA) (Liberati et al., 2009) statement and the Cochrane Handbook for the Systematic Review of interventions (details *via* http://training/cochrane.org/handbook).

### Data Sources and Search Strategy

We systematically searched the following databases from their inception to November 30, 2019: PubMed, MEDLINE, EMBASE, Cochrane Library, Scopus, Clarivate, and the Chinese databases of CNKI, WanFang, and VIP for relevant literature. The pre-established search terms consisted of three parts: strategies for GRED, THM treatment, and a specific filter for randomized controlled trials. Both Medical Subject Headings (MeSH) terms and text words were used for keywords. The detailed search strategies for each database are shown in **Supplementary Table S1**. No limitation was placed on language of article. Any omission of publications was remedied by manual retrieval. To obtain eligible trials, the reference lists of the included studies were checked for verification and further assessment.

### Study Selection

Following the PICOS (participants, interventions, comparisons, outcomes, and study design) criteria, two investigators (Yun-kai Dai, Yun-bo Wu) preliminarily screened the relevant titles and abstracts. Randomized, parallel-group clinical trials of THM for GERD were initially included. The full texts of these studies were then scanned for further evaluation. Briefly, participants over the age of 18 should meet the diagnosis criteria of GERD (DeVault and Castell, 2005). Any prescription of THM interventions and certain positive controls (PPIs, or gastrointestinal motility drugs (GMD), or combinations) were selected. Meanwhile, the sample size of each trial should not be less than 30/arm, and the duration of treatment should be at least 4 weeks. In order to obtain superior quality literature, works with a Jadad score above 1 was screened.

However, some participants or publications were excluded: pregnant women, patients with comorbidities such as severe cardio-cerebro-vascular diseases and cancers, published meeting abstracts, non-research articles and cross-over studies, and THM as positive control.

### Data Abstraction and Quality Evaluation

Using a prepiloted data extraction sheet, two researchers (Yun-kai Dai, Yun-bo Wu) independently conducted data abstraction and quality assessment. Relevant characteristics of participants (gender, age, and sample size), details of interventions and comparisons (regimen for treatment and duration), course of disease, primary outcomes (overall clinical efficacy and efficacy under gastroscope) and secondary outcomes (improvements of acid regurgitation and heartburn, reflux diagnostic questionnaire (RDQ) scores), side effects, and study design were extracted, as was the classification of GERD. Moreover, relevant missing information could be acquired if necessary through telephoning the corresponding authors.

On the basis of the Cochrane Collaboration Recommendations assessment tool (Savovic et al., 2018), the quality of the included trials was independently evaluated by two reviewers (Yun-kai Dai, Yun-bo Wu). Overall evaluation of methodological quality had seven aspects: (i) random sequence generation; (ii) allocation concealment; (iii) blinding of participants and personnel; (iv) blinding (or masking) of outcomes assessment; (v) incomplete outcome data; (vi) selective reporting; (vii) other bias. Disagreements were resolved by further discussion or negotiation. For the methodological quality attribute of each study, the value “high quality” (or low risk), “uncertain quality” (or unclear risk), or “low quality” (or high risk) was assigned to calculate the overall score, which ranged from 0 to 6 points (from worst to best methodological quality). In view of this, the distributions of the methodological qualities on different comparisons across the evidence network were assessed. In addition, the Grading of Recommendations Assessment, Development, and Evaluation (GRADE) profiler version 3.6 was used to analyze the overall evidence quality of this network analysis.

### Statistical Analysis

Evidence of direct and indirect multiple-intervention comparisons is obtained by network meta-analysis, and performing this analysis with the Bayesian framework can improve the accuracy of the results. WinBUGS version 1.4.3 (MRC Biostatistics Unit, Cambridge, UK), based on the Bayesian framework and the Markov chain Monte Carlo (MCMC) method, was used to assess and process research data a priori. We used non-informative uniform and normal prior distributions (Ades et al., 2006; Sutton et al., 2008) and three Markov chains to fit the model. Meanwhile, 50,000 simulation iterations and 10 thining intervals per chain were set to gain the posterior distributions of model parameters. The first 20,000 iterations were used for burn-in so as to eliminate the effects of initial value scaling, while the last 30,000 were applied to sampling. A relationship between direct and indirect multiple-intervention comparisons was drawn as a network figure using Stata version 13.0 software. The Brooks-Gelman-Rubin statistic was calculated to evaluate model convergence. The closer the potential scale reduction factor (PSRF) value was to 1, the better the convergence. Of course, a PSRF value of less than 1.2 was still acceptable. The node-splitting analysis was evaluated to test the consistency (Dias et al., 2010). If the *p*-value was greater than 0.05, a consistency model would be used. Otherwise, an inconsistency model was used. Accordingly, sensitivity analysis was used to test the source of heterogeneity. To summarize the probabilities for all interventions, the surface under the cumulative ranking curve (SUCRA) was used as a summary statistic for the cumulative ranking (Salanti et al., 2011). Based on the definition, the larger SUCRA scores are, the more effective the interventions are. In this study, the effect sizes of all outcomes were analyzed by a fixed or random effect model depending on indexes of statistical heterogeneity including the *p*-value and inconsistency index statistic (*I^2^*) (Higgins et al., 2003). Dichotomous outcomes were calculated using the odds ratio (OR) and 95% credible intervals (CrIs). Continuous variable data were evaluated using the mean difference (MD) and with their corresponding 95% CrIs.

## Results

### Study Identification and Selection

**Figure 1:**
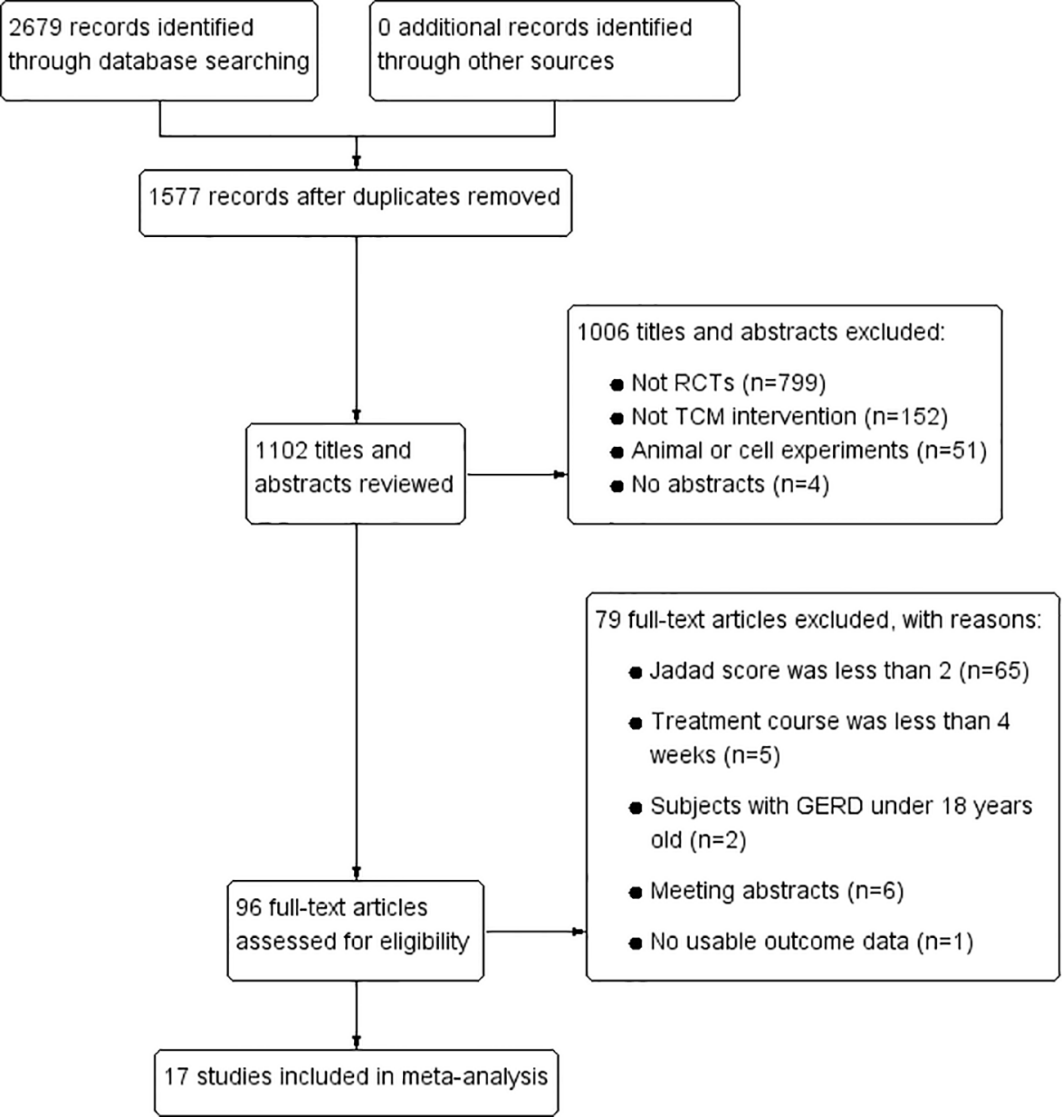
Flow diagram.

**Figure 2:**
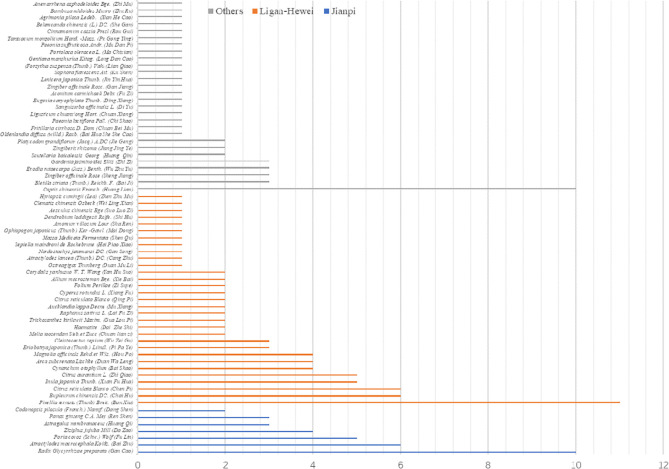
Usage frequency of the included herbs.

In total, 2679 articles were retrieved from the nine databases according to the corresponding search strategies. After removing duplicates and irrelevant publications, 17 randomized controlled trials (RCTs) including 1441 participants were selected for further quantitative analyses. A flow diagram of the specific retrieval process is shown in **Figure 1**. The baseline characteristics of the included trials are displayed in **Table 1**. The classification of herbal medicines and usage frequency of the included herbs can be found in **Table 2** and **Figure 2**. Accordingly, we could draw a rough conclusion that herbs with the function of regulating the liver and harmonizing the stomach (TCM jargon: Ligan Hewei) and invigorating spleen (TCM jargon: Jianpi) had higher frequencies among the included herbs.

**Table 1:** Characteristics of the studies included in the network analysis.

Study ID	Country/Affiliation of the first author	Classification of GERD	Sample Size		Age (years)	Course of disease(months)	Duration(weeks)	Intervention		Outcomes	Follow-up	Side effects
			EG (M/F)	CG (M/F)				EG	CG			
Li et al., 2019	China/Department of Spleen and Stomach Diseases, Beijing Hospital of TCM Affiliated to the Capital Medical University	N/A	38 (15/23)	36 (22/14)	E: 47. 42 ± 11.91 C: 49. 89 ± 11.82	E: 52.08 ± 51.60 C: 40.32 ± 34.44	6	Jianpi therapy	PPIs	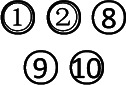	N/A	N/A
Shih et al., 2019	China (Taiwan)/Graduate Institute of Integrated Medicine, College of Chinese Medicine	N/A	40 (14/26)	37 (20/17)	E: 46.03 ± 13.88 C: 46.95 ± 13.42	N/A	4	Jianpi therapy	PPIs	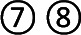	N/A	E: 1 for epigastric pain 1 for bitter taste 1 for fever sensation C:1 for abdominal pain 2 for epigastric pain 1 for worsened acid reflux
Liu et al., 2018	China/First People’s Hospital Affiliated to Shanghai Jiaotong University	RE	60 (24/36)	60 (32/28)	E: 47.55 ± 12.44 C: 46.03 ± 11.36	E: 85.08 ± 48.12 C: 83.76 ± 47.16	8	Ligan Hewei therapy	PPIs	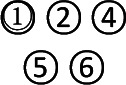	N/A	E: abdominal distension, nausea, insomnia, cold, rhinitis. C: nausea, diarrhea, tinnitus, swelling, aching of gums
Yang et al., 2018	China/Shanghai Fenglin Community Health Service Center of Xuhui District	N/A	43 (19/24)	42 (21/21)	E: 46.63 ± 13.80 C: 49.19 ± 11.75	E: 10.95 ± 2.69 C: 10.62 ± 3.59	8	Jianpi therapy	PPIs	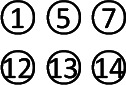	N/A	N/A
Li, 2018	China/Chengdu University of TCM	N/A	30 (17/13)	30 (18/12)	E: 2.13 ± 14.08 C: 52.47 ± 13.71	E: 37.72 ± 7.82 C: 38.88 ± 7.12	8	Ligan Hewei therapy	PPIs +GMD	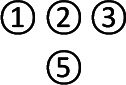	N/A	N/A
Wang, 2018	China/Chengdu University of TCM	RE	30 (14/16)	30 (15/15)	E: 52.58 ± 6.22 C: 53.52 ± 7.19	E: 19.77 ± 8.11 C: 20.07 ± 8.26	8	Jianpi therapy	PPIs	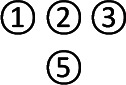	N/A	N/A
Huang et al., 2017	China/Department of Spleen and Stomach Diseases, Yancheng Hospital of TCM	NERD	43 (25/18)	43 (23/20)	E: 40.50 ± 9.40 C: 41.70 ± 11.20	E: 76.80 ± 64.80 C: 80.40 ± 69.60	4	Ligan Hewei therapy	PPIs	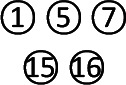	N/A	E: diarrhea
Zhang et al., 2017	China/The Second Clinical Medical College, Henan University of TCM	N/A	40 (25/15)	40 (19/21)	E: 53.30 ± 5.10 C: 55.60 ± 5.60	N/A	8	Ligan Hewei therapy	PPIs	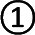	N/A	N/A
Wu and Ge, 2016	China/Xiangcheng District TCM Hospital of Suzhou City	N/A	65 (30/35)	62 (28/34)	E: 45.80 ± 13.10 C: 46.30 ± 12.70	E: 20.10 ± 16.80 C: 18.30 ± 16.20	8	Ligan Hewei therapy	PPIs +GMD	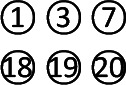	N/A	N/A
Xue, 2015	China/Shandong University of TCM	N/A	30 (10/20)	30 (11/19)	E: 48.90 ± 7.50 C: 49.20 ± 8.10	E: 45.30 ± 3.80 C: 46.20 ± 5.10	12	Ligan Hewei therapy	PPIS +GMD	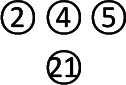	N/A	E: N/A C: 2 for nausea
Sakata et al., 2014	Japan/Department of Internal Medicine and Gastroenterology, Saga Medical School	NERD	52 (17/35)	43 (8/35)	E: 72.10 C: 73.40	N/A	8	Jianpi therapy	PPIs	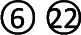	N/A	N/A
Wang, 2014	China/Shanxi University of Chinese Medicine	RE	33 (20/13)	34 (22/12)	E: 45.12 ± 6.32 C: 46.78 ± 9.77	E: 42.72 ± 27.60 C: 49.20 ± 19.80	8	Ligan Hewei therapy +PPIs	PPIs	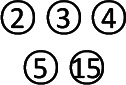	N/A	E:3 for slight nausea. C: 6 for nausea, poor appetite, and diarrhea.
Cheng et al., 2013	China/Yueyang Hospital of Integrative Chinese and Western Medicine Affiliated to Shanghai University of TCM	NERD	30 (12/18)	30 (7/23)	E: 50.47 ± 11.62 C: 46.63 ± 12.40	N/A	8	Ligan Hewei therapy	PPIs	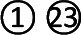	N/A	N/A
Tominaga et al., 2012	Japan/Department of Gastroenterology, Osaka City University Graduate School of Medicine	N/A	50 (20/30)	51 (17/34)	E: 63.60 C: 64.50	N/A	4	Jianpi therapy	PPIs	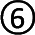	N/A	N/A
Hao and Zhang, 2012	China/Shanxi Medical University	N/A	40 (24/16)	40 (23/17)	E: 59.40 ± 7.80 C: 58.50 ± 11.40	N/A	4	Ligan Hewei therapy	GMD	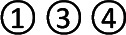	2 months	N/A
Zhang, 2012	China/Nanjing University of Chinese Medicine	N/A	55 (31/24)	54 (30/24)	E: 49.20 ± 13.10 C: 46.80 ± 12.40	N/A	8	Jianpi therapy +PPIs	PPIs	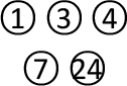	1 month	N/A
Zhu et al., 2010	China/Shanghai Hospital of TCM Attached to Shanghai University of TCM	N/A	50 (16/34)	50 (14/36)	E: 51.48 C: 48.98	E: 34.80 C: 44.04	8	Ligan Hewei therapy	PPIs	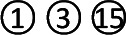	N/A	N/A

Annotations: E, experimental group; C, control group; N/A, not applicable; TCM, traditional Chinese medicine; GERD, gastroesophageal reflux disease; NERD, non-erosive reflux disease; RE, reflux esophagitis; M, male; F, female; PPIs, proton pump inhibitors; GMD, gastrointestinal motility drugs; 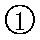, overall clinical efficacy; 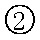, TCM symptom scores (belching, acid regurgitation, heartburn, sternalgia); 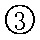, efficacy under gastroscope; 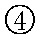, recurrence rate; 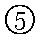, adverse effect rate; 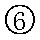, FSSG questionnaire frequency scale for the symptoms of GERD; 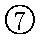, Reflux Disease Questionnaire (RDQ); 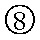, GERD Questionnaire scores (GerdQ); 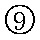, The short-form health survey questionnaire (SF 36); 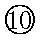, 24-hour esophageal pH monitoring; 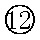, GERD Health-Related Quality of Life Questionnaire (GERD-HRQL); 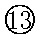, Pittsburgh Sleep Quality Index (PSQI); 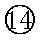, Hospital Anxiety and Depression Scale (HADS); 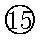, clinical symptoms scores; 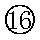, Serum Ghrelin, LPO level; 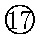, total clinical symptoms score; 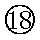, scores of gastroscopy evaluation; 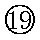, pressure of upper esophageal sphincter (UES) and lower esophageal sphincter (LES); 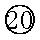, scores of the pattern of depressed liver and stomach qi transforming into fire; 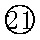, scores of anxiety and depression; 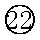, Gastrointestinal Symptom Rating Scale (GSRS); 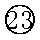, total scores of symptoms and physical signs; 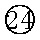, efficacy of main symptoms (belching, regurgitation, heartburn, sternalgia).

**Table 2:** The ingredients of each formula in the included trials.

Author	Ingredients of each formula			
Li et al., 2019	*Codonopsis pilosula (Franch.) Nannf.* (Dang Shen)	*Allium macrostemon Bge.* (Xie Bai)	*Atractylodes lancea (Thunb.) DC.* (Cang Zhu)	*Trichosanthes kirilowii Maxim.* (Gua Lou Pi)
	*Coptis chinensis Franch.* (Huang Lian)	*Evodia cuspidata K.Schum.* (Wu Zhu Yu)	*Aucklandia lappa DC.* (Mu Xiang)	*Eugenia abbreviata Urb.* (Ding Xiang)
	*Citrus reticulata Blanco* (Qing Pi)	*Aesculus chinensis Bge* (Suo Luo Zi)	*Pinellia ternata (Thunb) Breit.* (Ban Xia)	*Citrus mitis Blanco* (Chen Pi)
	*Bambusa tuldoides Munro* (Zhu Ru)			
Shih et al., 2019	*Evodia cuspidata K.Schum.* (Wu Zhu Yu)	*Panax ginseng C.A. Mey* (Ren Shen)	*Ziziphus jujuba Mill* (Da Zao)	*Zingiber Officinale Roscoe* (Sheng Jiang)
Liu et al., 2018	*Dendrobium loddigesii Rolfe.* (Shi Hu)	*Anemarrhena asphodeloides Bge.* (Zhi Mu)	*Coptis chinensis Franch.* (Huang Lian)	*Sophora flavescens Ait.* (Ku Shen)
	*Poria cocos (Schw.) Wolf* (Fu Lin)	*Atractylodes macrocephala Koidz.* (Bai Zhu)	*Bletilla striata (Thunb.) Reichb. F.* (Bai Ji)	*Astragalus mongholicus Bunge* (Huang Qi)
	*Portulaca oleracea L.* (Ma Chi Xian)	*Citrus aurantium L.* (Zhi Qiao)	*Clematis chinensis Osbeck* (Wei Ling Xian)	*Iris domestica (L.) Goldblatt & Mabb.* (She Gan)
	*Forsythia suspensa (Thunb.) Vahl* (Lian Qiao)	*Sanguisorba officinalis L.* (Di Yu)		
Yang et al., 2018	*Atractylodes macrocephala Koidz.* (Bai Zhu)	*Eriobotrya japonica (Thunb.) Liindl.* (Pi Pa Ye)	*Platycodon grandiflorum (Jacq.) A.DC* (Jie Geng)	
Li, 2018	*Bupleurum chinensis DC.* (Chai Hu)	*Citrus aurantium L.* (Zhi Qiao)	*Paeonia lactiflora Pall.* (Chi Shao)	*Radix Glycyrrhizae preparata* (Gan Cao)
	*Astragalus mongholicus Bunge* (Huang Qi)	*Cyperus rotundus L.* (Xiang Fu)	*Massa Medicata Fermentata* (Shen Qu)	*Pinellia ternata (Thunb) Breit.* (Ban Xia)
	*Corydalis yanhusuo W.T. Wang* (Yan Hu Suo)	*Allium macrostemon Bge.* (Xie Bai)	*Trichosanthes kirilowii Maxim.* (Gua Lou Pi)	*Inula japonica Thunb.* (Xuan Fu Hua)
	*Raphanus raphanistrum subsp. sativus (L.) Domin* (Lai Fu Zi)	*Scutellaria baicalensis Georg* (Huang Qin)		
Wang, 2018	*Astragalus mongholicus Bunge* (Huang Qi)	*Panax ginseng C.A. Mey* (Ren Shen)	*Cinnamomum cassia Presl* (Rou Gui)	*Paeonia lactiflora Pall.* (Bai Shao)
	*Zingiberis rhizoma* (Gan Jiang)	*Ziziphus jujuba Mill* (Da Zao)	*Radix Glycyrrhizae preparata* (Gan Cao)	*Aconitum carmichaeli Debx* (Fu Zi)
	*Pinellia ternata (Thunb) Breit.* (Ban Xia)			
Huang et al., 2017	*Coptis chinensis Franch.* (Huang Lian)	*Corydalis yanhusuo W.T. Wang* (Yan Hu Suo)	*Citrus mitis Blanco* (Chen Pi)	*Cleistocactus sepium* (Wu Zei Gu)
	*Pinellia ternata (Thunb) Breit.* (Ban Xia)	*Fritillaria cirrhosa D. Dom* (Chuan Bei Mu)	*Magnolia officinals Rehd.et Wils.* (Hou Po)	*Inula japonica Thunb.* (Xuan Fu Hua)
	*Oldenlandia diffusa (Willd.) Roxb.* (Bai Hua She She Cao)	*Lonicera japonica Thunb.* (Jin Yin Hua)	*Ophiopogon japonicus (Thunb.) Ker-Gawl.* (Mai Dong)	*Radix Glycyrrhizae preparata* (Gan Cao)
Zhang et al., 2017	*Citrus mitis Blanco* (Chen Pi)	*Pinellia ternata (Thunb) Breit.* (Ban Xia)	*Bletilla striata (Thunb.) Reichb. F.* (Bai Ji)	*Sepiella maindroni de Rochebrune* (Hai Piao Xiao)
	*Fritillaria cirrhosa D. Dom* (Chuan Bei Mu)	*Arca subcrenata Lischke* (Duan Wa Leng)	*Ostreagigas Thunberg* (Duan Mu Li)	*Coptis chinensis Franch.* (Huang Lian)
	*Evodia cuspidata K.Schum.* (Wu Zhu Yu)	*Nardostachys jatamansi DC.* (Gan Song)		
Wu and Ge, 2016	*Coptis chinensis Franch.* (Huang Lian)	*Evodia cuspidata K.Schum.* (Wu Zhu Yu)	*Pinellia ternata (Thunb) Breit.* (Ban Xia)	*Magnolia officinals Rehd.et Wils.* (Hou Po)
	*Poria cocos (Schw.) Wolf* (Fu Lin)	*Zingiber Officinale Roscoe* (Sheng Jiang)	*Folium Perillae* (Zi Su Ye)	*Inula japonica Thunb.* (Xuan Fu Hua)
	*Gardenia jasminoides Ellis* (Zhi Zi)	*Melia azedarach L.* (Chuan Lian Zi)	*Cyperus rotundus L.* (Xiang Fu)	*Gentiana manshurica Kitag.* (Long Dan Cao)
	*Bupleurum chinensis DC.* (Chai Hu)	*Radix Glycyrrhizae preparata* (Gan Cao)		
Xue, 2015	*Bupleurum chinensis DC.* (Chai Hu)	*Coptis chinensis Franch.* (Huang Lian)	*Evodia cuspidata K.Schum.* (Wu Zhu Yu)	*Paeonia lactiflora Pall.* (Bai Shao)
	*Citrus aurantium L.* (Zhi Qiao)	*Atractylodes macrocephala Koidz.* (Bai Zhu)	*Pinellia ternata (Thunb) Breit.* (Ban Xia)	*Poria cocos (Schw.)Wolf* (Fu Lin)
	*Aucklandia lappa DC.* (Mu Xiang)	*Amomum villosum Lour.* (Sha Ren)	*Folium Perillae* (Zi Su Ye)	*Eriobotrya japonica (Thunb.) Liindl.* (Pi Pa Ye)
	*Arca subcrenata Lischke* (Duan Wa Leng)	*Radix Glycyrrhizae preparata* (Gan Cao)		
Sakata et al., 2014	*Atractylodes macrocephala Koidz.* (Bai Zhu)	*Panax ginseng C.A. Mey* (Ren Shen)	*Pinellia ternata (Thunb) Breit.* (Ban Xia)	*Poria cocos (Schw.) Wolf* (Fu Lin)
	*Ziziphus jujuba Mill* (Da Zao)	*Citrus mitis Blanco* (Chen Pi)	*Radix Glycyrrhizae preparata* (Gan Cao)	*Zingiberis rhizoma* (Gan Jiang)
Wu and Ge, 2016	*Bupleurum chinensis DC.* (Chai Hu)	*Coptis chinensis Franch.* (Huang Lian)	*Paeonia suffruticosa Andr.* (Mu Dan Pi)	*Paeonia lactiflora Pall.* (Bai Shao)
	*Fritillaria cirrhosa D. Dom* (Chuan Bei Mu)	*Pinellia ternata (Thunb) Breit.* (Ban Xia)	*Citrus reticulata Blanco* (Qing Pi)	*Evodia cuspidata K.Schum.* (Wu Zhu Yu)
	*Cleistocactus sepium* (Wu Zei Gu)	*Magnolia officinals Rehd.et Wils.* (Hou Po)	*Taraxacum mongolicum Hand. -Mazz.* (Pu Gong Ying)	*Radix Glycyrrhizae preparata* (Gan Cao)
Cheng et al., 2013	*Inula japonica Thunb.* (Xuan Fu Hua)	*Haematite* (Dai Zhe Shi)	*Bupleurum chinensis DC.* (Chai Hu)	*Citrus aurantium L.* (Zhi Qiao)
	*Gardenia jasminoides Ellis* (Zhi Zi)	*Melia azedarach L.* (Chuan Lian Zi)	*Coptis chinensis Franch.* (Huang Lian)	*Evodia cuspidata K.Schum.* (Wu Zhu Yu)
	*Zingiber Officinale Roscoe* (Sheng Jiang)	*Arca subcrenata Lischke* (Duan Wa Leng)	*Radix Glycyrrhizae preparata* (Gan Cao)	
Tominaga et al., 2012	*Atractylodes macrocephala Koidz.* (Bai Zhu)	*Panax ginseng C.A. Mey* (Ren Shen)	*Pinellia ternata (Thunb) Breit.* (Ban Xia)	*Poria cocos (Schw.) Wolf* (Fu Lin)
	*Ziziphus jujuba Mill* (Da Zao)	*Citrus mitis Blanco* (Chen Pi)	*Radix Glycyrrhizae preparata* (Gan Cao)	*Zingiberis rhizoma* (Gan Jiang)
Hao and Zhang, 2012	*Bupleurum chinensis DC.* (Chai Hu)	*Paeonia lactiflora Pall.* (Bai Shao)	*Citrus aurantium L.* (Zhi Qiao)	*Citrus mitis Blanco* (Chen Pi)
	*Conioselinum chinense (L.) Britton, Sterns & Poggenb.* (Chuan Xiong)	*Gardenia jasminoides Ellis* (Zhi Zi)	*Coptis chinensis Franch.* (Huang Lian)	*Pinellia ternata (Thunb) Breit.* (Ban Xia)
	*Evodia cuspidata K.Schum.* (Wu Zhu Yu)	*Raphanus raphanistrum subsp. sativus (L.) Domin* (Lai Fu Zi)	*Hyriopsis cumingii (Lea)* (Zhen Zhu Mu)	*Radix Glycyrrhizae preparata* (Gan Cao)
Zhang, 2012	*Coptis chinensis Franch.* (Huang Lian)	*Evodia cuspidata K.Schum.* (Wu Zhu Yu)	*Codonopsis pilosula (Franch.) Nannf.* (Dang Shen)	*Inula japonica Thunb.* (Xuan Fu Hua)
	*Haematite* (Dai Zhe Shi)	*Scutellaria baicalensis Georg* (Huang Qin)	*Cleistocactus sepium* (Wu Zei Gu)	*Fritillaria cirrhosa D. Dom* (Chuan Bei Mu)
	*Agrimonia pilosa Ledeb.* (Xian He Cao)	*Bletilla striata (Thunb.) Reichb. F.* (Bai Ji)	*Citrus aurantium L.* (Zhi Qiao)	*Magnolia officinals Rehd.et Wils.* (Hou Po)
Zhu et al., 2010	*Eriobotrya japonica (Thunb.) Liindl.* (Pi Pa Ye)	*Platycodon grandiflorum (Jacq.) A.DC* (Jie Geng)	*Atractylodes macrocephala Koidz.* (Bai Zhu)	

Annotations: Italics are Latin terms for herbs. Non-italics are Chinese pinyin for herbs.

### Risk of Bias Evaluation

**Figure 3:**
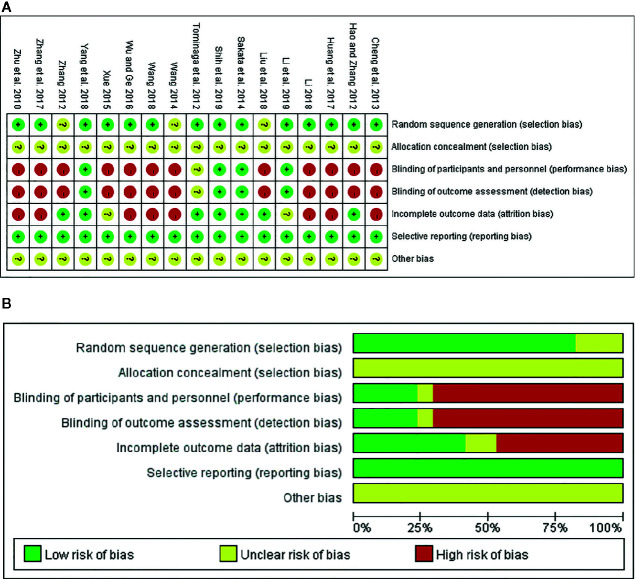
Risk of bias. **(A)** Summary. **(B)** Graph.

On the basis of the Cochrane Collaboration Recommendations evaluation tools (Savovic et al., 2014), the quality of the included RCTs was assessed. Of all the studies, 82.35% (14/17) gave a specific description of the random-allocation process, such as the use of a random number table or a computer-generated randomization list. The others only used the word “randomization” without any explanation. Because of insufficient information about allocation concealment, all included trials were judged as of “unclear risk.” In performance bias, only four studies (23.53%) described double or single blinding. As for detection bias, 13 trials (76.47%) either could not be blinded or it was unclear whether they had been. In addition, eight RCTs (47.06%) were at low risk of attrition bias because they provided detailed explanations or statistical estimations of dropout rates. However, two trials (11.76%) failed to provide adequate information for the judgment of missing data risk. Moreover, there was insufficient information on other risks for all 17 studies. In sum, among all trials, 4 were viewed as low risk, 2 as unclear risk, and 11 as high risk. A detailed quality evaluation is shown in **Figures 3A, B**.

### Network Evidence

**Figure 4:**
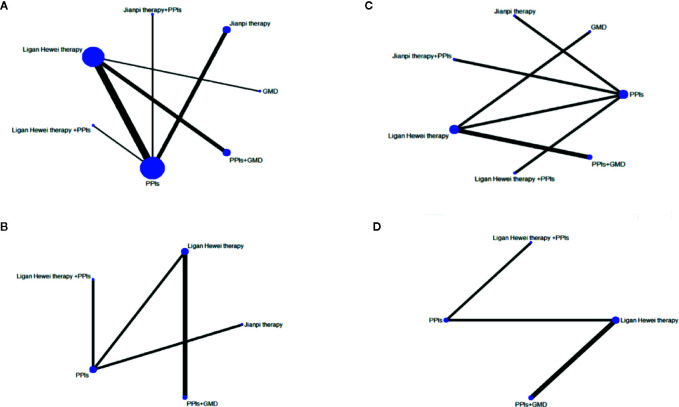
Network evidence diagrams of four endpoints. **(A)** Overall clinical efficacy. **(B)** Improvement of acid regurgitation. **(C)** Efficacy under gastroscope. **(D)** Improvement of heartburn.

This study included seven regimens as follows: Ligan Hewei therapy, Ligan Hewei therapy+PPIs, Jianpi therapy, Jianpi therapy+PPIs, PPIs, PPIs+GMD, and GMD. The results of the network analysis suggested that the number of GERD patients treated with PPIs was the largest, followed by Ligan Hewei therapy and then Jianpi therapy, while the number of GERD patients treated with Ligan Hewei therapy+PPIs was the smallest (**Figure 4**).

### Major Results of Network Analysis

In this study, there were 14 publications reporting overall clinical efficacy, 7 reporting the improvement of gastroesophageal mucosal lesions when viewed under a gastroscope (namely efficacy under gastroscope), 5 reporting improvement of acid regurgitation, and 4 reporting heartburn improvement. As shown in **Table S2**, the results of node-splitting between the direct and indirect effects showed no inconsistency for the four outcomes (P>0.05). Meanwhile, the PSRF value with 1 or 1.01 indicated good convergence and a stable result. Therefore, a model of consistency was built. As displayed in **Table 3**, Ligan Hewei therapy was significantly better overall clinical efficacy than PPIs (OR 2.36, 95%CrIs 1.16 to 5.24) and GMD+PPIs (OR 2.99, 95%CrIs 1.09 to 8.60). For the improvement of acid regurgitation, Ligan Hewei therapy+PPIs (MD -1.51, 95%CrIs -3.45 to 0.48), Ligan Hewei therapy (MD -1.07, 95%CrIs -3.10 to 0.93), and PPIs (MD -0.93, 95%CrIs -2.47 to 0.56) were superior to Jianpi therapy, Ligan Hewei therapy+PPIs (MD -0.58, 95%CrIs -1.84 to 0.74) was better than PPIs, and PPIs+GMD was superior to Jianpi therapy (MD -1.25, 95%CrIs -3.49 to 0.92) and Ligan Hewei therapy (MD -0.18, 95%CrIs -1.13 to 0.71). For heartburn improvement, Ligan Hewei therapy+PPIs (MD -0.76, 95%CrIs -1.73 to 0.24), PPIs+GMD (MD -0.34, 95%CrIs -1.56 to 0.85), and Ligan Hewei therapy (MD -0.19, 95%CrIs -1.18 to 0.78) were better than PPIs, and Ligan Hewei therapy+PPIs (MD -0.56, 95%CrIs -1.91 to 0.83) and PPIs+GMD (MD -0.15, 95%CrIs -0.84 to 0.52) were superior to Ligan Hewei therapy.

**Table 3:** Odds ratios (OR) or mean difference and 95% credible intervals (CrIs) of seven regimes with four endpoints.

OR/MD (95%CrIs)						
Overall clinical efficacy						
**GMD**	1.09 (0.15, 9.61)	3.43 (0.37, 35.53)	5.37 (0.35, 113.24)	3.29 (0.55, 21.69)	3.27 (0.28, 41.96)	1.38 (0.19, 10.36)
0.91 (0.10, 6.75)	**PPIs + GMD**	3.06 (0.60, 15.96)	4.71 (0.46, 72.18)	**2.99 (1.09, 8.60)**	2.95 (0.39, 24.96)	1.26 (0.36, 4.54)
0.29 (0.03, 2.67)	0.33 (0.06, 1.68)	**Jianpi therapy**	1.49 (0.17, 19.80)	0.98 (0.25, 3.25)	1.00 (0.14, 5.88)	0.42 (0.14, 1.12)
0.19 (0.01, 2.87)	0.21 (0.01, 2.17)	0.67 (0.05, 5.99)	**Jianpi therapy + PPIs**	0.65 (0.05, 5.23)	0.63 (0.04, 7.79)	0.28 (0.02, 1.85)
0.30 (0.05, 1.82)	**0.33 (0.12, 0.92)**	1.02 (0.31, 3.98)	1.54 (0.19, 19.54)	**Ligan Hewei therapy**	1.00 (0.18, 5.67)	**0.42 (0.19, 0.86)**
0.31 (0.02, 3.51)	0.34 (0.04, 2.59)	1.00 (0.17, 7.36)	1.59 (0.13, 27.40)	1.00 (0.18, 5.62)	**Ligan Hewei therapy + PPIs**	0.43 (0.09, 1.92)
0.73 (0.10, 5.14)	0.79 (0.22, 2.79)	2.40 (0.90, 7.38)	3.60 (0.54, 40.86)	**2.36 (1.16, 5.24)**	2.35 (0.52, 11.22)	**PPIs**
						
Efficacy under gastroscope						
**GMD**	1.65 (0.10, 26.24)	11.61 (0.25, 568.68)	51.90 (0.92, 3257.59)	7.11 (0.74, 68.82)	26.28 (0.58, 1280.91)	7.98 (0.31, 175.41)
0.61 (0.04, 9.72)	**PPIs + GMD**	6.89 (0.21, 254.10)	31.25 (0.78, 1584.93)	4.25 (0.88, 22.93)	16.30 (0.54, 600.63)	4.83 (0.33, 75.59)
0.09 (0.00, 3.99)	0.15 (0.00, 4.80)	**Jianpi therapy**	4.24 (0.18, 128.60)	0.61 (0.03, 13.90)	2.29 (0.11, 48.17)	0.69 (0.07, 5.85)
0.02 (0.00, 1.09)	0.03 (0.00, 1.27)	0.24 (0.01, 5.64)	**Jianpi therapy + PPIs**	0.14 (0.00, 3.71)	0.55 (0.02, 14.63)	0.17 (0.01, 1.87)
0.14 (0.01, 1.35)	0.24 (0.04, 1.14)	1.64 (0.07, 37.39)	7.19 (0.27, 247.10)	**Ligan Hewei therapy**	3.73 (0.18, 87.69)	1.15 (0.12, 10.03)
0.04 (0.00, 1.72)	0.06 (0.00, 1.85)	0.44 (0.02, 8.98)	1.83 (0.07, 56.23)	0.27 (0.01, 5.44)	**Ligan Hewei therapy + PPIs**	0.30 (0.03, 2.61)
0.13 (0.01, 3.18)	0.21 (0.01, 3.07)	1.44 (0.17, 13.42)	6.06 (0.53, 99.03)	0.87 (0.10, 8.10)	3.37 (0.38, 30.87)	**PPIs**
						
Improvement of acid regurgitation						
**PPIs + GMD**	**-1.25 (-3.49, 0.92)**	**-0.18 (-1.13, 0.71)**	0.26 (-1.81, 2.18)	-0.31 (-1.93, 1.21)		
1.25 (-0.92, 3.49)	Jianpi therapy	1.07 (-0.93, 3.10)	1.51 (-0.48, 3.45)	0.93 (-0.56, 2.47)		
0.18 (-0.71, 1.13)	**-1.07 (-3.10, 0.93)**	Ligan Hewei therapy	0.44 (-1.40, 2.19)	-0.13 (-1.46, 1.13)		
-0.26 (-2.18, 1.81)	**-1.51 (-3.45, 0.48)**	-0.44 (-2.19, 1.40)	Ligan Hewei therapy + PPIs	-0.58 (-1.84, 0.74)		
0.31 (-1.21, 1.93)	**-0.93 (-2.47, 0.56)**	0.13 (-1.13, 1.46)	0.58 (-0.74, 1.84)	PPIs		
						
Improvement of heartburn						
**PPIs + GMD**	**-0.15 (-0.84, 0.52)**	0.41 (-1.16, 1.92)	**-0.34 (-1.56, 0.85)**			
0.15 (-0.52, 0.84)	**Ligan Hewei therapy**	0.56 (-0.83, 1.91)	**-0.19 (-1.18, 0.78)**			
-0.41 (-1.92, 1.16)	-0.56 (-1.91, 0.83)	**Ligan Hewei therapy + PPIs**	**-0.76 (-1.73, 0.24)**			
0.34 (-0.85, 1.56)	0.19 (-0.78, 1.18)	0.76 (-0.24, 1.73)	PPIs			

OR or MD and 95%CrIs below the treatments should be read from row to column while those above should be read from column to row. PPIs, proton pump inhibitors; GMD, gastrointestinal motility drugs. Bolded data indicated P < 0.05.

### SUCRA Value

**Figure 5:**
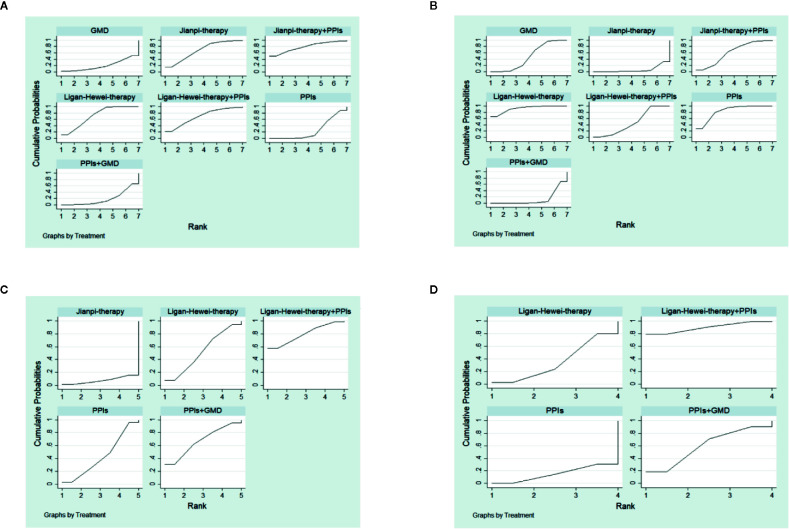
The surface under the cumulative ranking curve (SUCRA) plots of four endpoints. **(A)** Overall clinical efficacy. **(B)** Efficacy under gastroscope.**(C)** Improvement of acid regurgitation. **(D)** Improvement of heartburn.

The SUCRA-based rankings of all treatments are displayed in **Figure 5**. In terms of overall clinical efficacy, Jianpi therapy+PPIs (78.7%) ranked first, followed by Ligan Hewei therapy (71.1%), Ligan Hewei therapy+PPIs (69.2%), Jianpi therapy (67.8%), PPIs (25.4%), GMD (19.6%), and PPIs+GMD (18.3%). However, viewed as a whole, the SUCRA value of efficacy under gastroscope was higher for Ligan Hewei therapy (92.0%) than for any other treatments. As for the improvement of acid regurgitation and heartburn, the highest SUCRA value was found for Ligan Hewei therapy+PPIs (acid regurgitation: 79.4%; heartburn: 90.0%).

### Sensitivity Analysis

**Figure 6:**
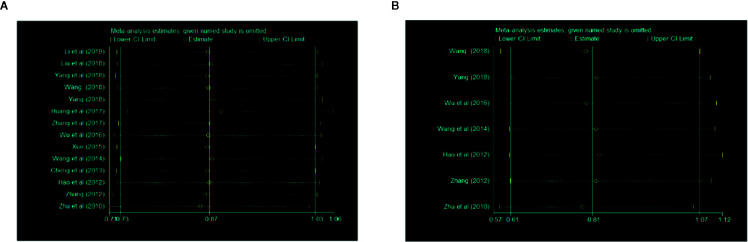
Sensitivity analyses of **(A)** overall clinical efficacy and **(B)** efficacy under gastroscope.

A sensitivity analysis was conducted through omitting studies one by one. The result of this analysis showed that there were no significant differences in overall clinical efficacy and efficacy under gastroscope (**Figure 6**).

### GRADE Evidence of Quality

**Figure 7:**
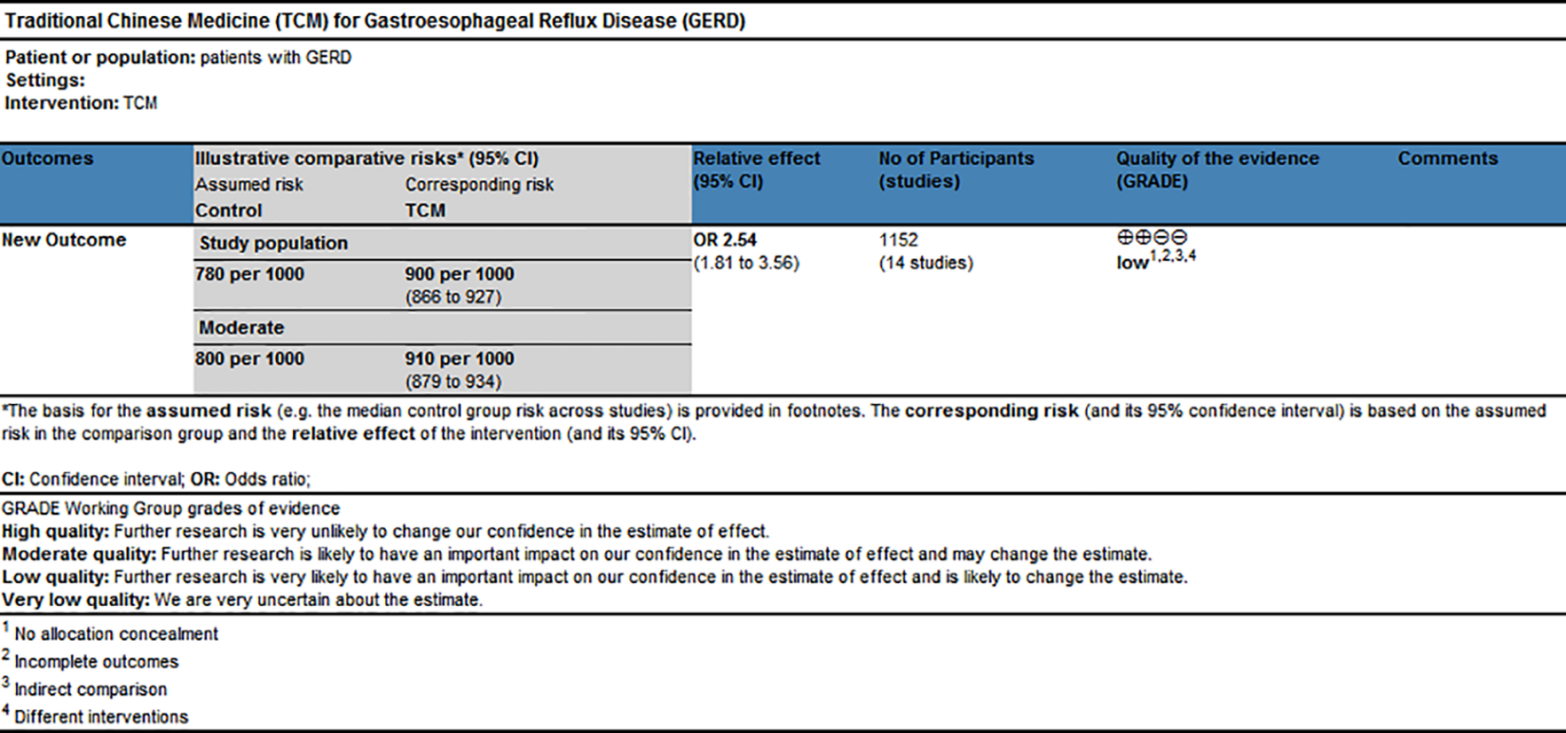
GRADE quality grading evaluation.

GRADE profiler software, which includes the elements of GRADE criteria such as study design, risk of bias, inconsistency, indirectness, imprecision, and publication bias, was used to rate the quality of evidence and grade strength of recommendations for this network meta-analysis. The results shown in **Figure 7** suggested that the evidence quality of overall clinical efficacy was “Low,” which could be related to high risk of bias and indirectness within RCTs.

## Discussion

Network meta-analysis is used to analyze studies with multiple interventions and provide rankings for them (Naci et al., 2014). Our findings from the comprehensive network analyses demonstrated the overall synthesis of data for currently available GERD treatments in terms of different THM. Regarding the usage frequency of each herb, a rough conclusion was drawn that herbs with the function of Ligan Hewei and Jianpi were used more frequently among the included herbs. In terms of outcomes, we found that Jianpi therapy+PPIs ranked first in overall clinical efficacy. Ligan Hewei therapy might be a better choice for healing gastroesophageal mucosal lesions according to gastroscope observations. In addition, in the improvement of acid regurgitation and heartburn, Ligan Hewei therapy+PPIs was superior to other interventions. Therefore, Ligan Hewei therapy and Jianpi therapy could be promising complementary and alternative therapies in the management of GERD, which potentially provides TCM practitioners with more suggestions and guidance in clinical decisions, as well as for treatments based on syndrome differentiation.

The pathogenesis of GERD is poorly understood so far. Currently, some acknowledged potential mechanisms are not only involved in hiatal hernia (Dore et al., 2016), anti-reflux barrier dysfunction (Xie et al., 2017), esophageal inflammation (Dunbar et al., 2016), and transient lower esophageal sphincter relaxation (TLESR) (Banovcin et al., 2016) but have also been associated with psychological factors (Baker et al., 1995; Wright et al., 2005) and obesity (Nadaleto et al., 2016). However, in the modern pharmacological field, complementary and alternative medicine (CAM), especially TCM, could potentially intervene in these mechanisms. A clinical study showed that wu chu yu tang (affiliated to Jianpi therapy) could improve the symptoms of GERD through anti-inflammation, antioxidant activity, acid suppression, reduction in pepsin secretion, and mucosal protection (Shih et al., 2019). In the treatment of gastrointestinal (GI) reflux diseases, another study indicated that Wendan decoction (WDD, affiliated with Ligan Hewei therapy) could reduce unhealthy emotions in patients *via* normalizing behaviors and up-regulating orexin-A, orexin receptor 1, and leptin and its receptor in the brain (Ling et al., 2015). Additionally, WDD could solve phlegm-related problems and recover GI homeostasis through dual action on acid and bile secretion (Xu et al., 2015). Meanwhile, acupuncture regulating qi based on the compatibility of the five meridians (affiliated to Ligan Hewei therapy) could also play an important role in treating GERD with disharmony between liver and stomach syndrome, whose mechanisms were possibly related to its regulation in the neuro-endocrine-immune system, thereby alleviating TLESR, promoting GI motility, suppressing acid secretion, and protecting gastric mucosa (Pan et al., 2017). Besides, acupoint drug finger pressing, based on the TCM theory of Jianpi therapy, also showed good therapeutic effects on GERD, which is probably attributable to lower esophageal sphincter pressure promotion and decrease in acid reflux in esophagus, as well as the improvement of coordination of gastroesophageal movement (Xie et al., 2007). In sum, CAM, especially TCM, may be multi-target treatments of GERD that are worth studying further *in vitro* and vivo.

Generally speaking, non-randomized trials are susceptible to many biases that affect the weaker forms of evidence. However, in RCTs, certain deficits in their design, conduct, analysis, and reporting may result in bias (Savovic et al., 2018). In this study, the methodological quality of the included trials was generally moderate, and the quality level of evidence for overall clinical efficacy, according to GRADE evidence classification, was “Low.” Analyzing from the above two results, the potential risk of bias in our study was possibly rooted in three aspects. First, there were 13/17 (76.47%) RCTs in which blinding as not implemented, which may lead to the occurrence of performance and detection biases. Next, due to the absence of allocation concealment in all of the included studies, the subjects could easily recognize which treatment they were allocated to, inevitably resulting in selection bias. Last, although 8/17 (47.06%) studies reported detailed withdrawals or dropouts, another 2 (11.76%) failed to provide an adequate explanation for missing data, which may also increase the risk of attrition bias.

In network analysis, consistency is characterized as a single comparison of the relationship between direct and indirect sources of evidence (Madan et al., 2011). When consistency is not good in a statistical analysis, it could be short of transitivity. In our study, for primary and secondary outcomes, based on the “node-splitting” method, it showed good convergence and strong stability, thereby further proving high reliability in our results. Nevertheless, clinical heterogeneity, for example, regarding the improvement of symptoms (acid regurgitation and heartburn), which were assessed by the standard excessively subjective judgments by doctors or patients, cannot be ruled out. Also, it should be taken into consideration that overall clinical efficacy and efficacy under gastroscope were described as comprehensive evaluation of the improvement of both many types of GERD symptoms and histopathological changes of gastroesophageal mucosa.

There are several potential limitations to our study. First, the included studies were only in Chinese and Japanese. Evidence with this geographically limited distribution needs more multicenter and large-scale research around the world to support it. Second, discrepancies in traditional herbal medicines (specifically, the interventions mentioned in our study) may exist because of their source and preparation, which could influence the strength of the evidence. Third, missing data could pose a threat to the validity of RCTs because it means that the observed outcomes of an RCT are not representative of all RCTs in the trial. Meanwhile, there was no corresponding evidence to verify its impact on the overall results in our study. Fourth, there was no unified criterion for the classification of interventions. Accordingly, we categorized them by the functions of herbs or prescriptions in the literature. Last, high quality of RCTs plays a key role in the production of optimal sources of evidence.

Therefore, we are looking forward to further standardized research and superior methodology, such as multicenter, large sample sizes, and well-designed (including the implementation of allocation concealment and blinding) RCTs to update and perfect the current body of evidence. Furthermore, strictly following the Consolidated Standards of Reporting Trials (CONSORT) or Standards for Reporting Interventions in Controlled Trials (STRICTA) statement is also essential to improve the reporting quality of future research.

## Conclusion

Evidence from this network analysis indicates that Ligan Hewei therapy and Jianpi therapy could be the most suitable complementary and alternative interventions for GERD. According to different evaluation outcomes, Jianpi therapy+PPIs could be an optimum treatment in terms of overall clinical efficacy. Ligan Hewei therapy might be suitable for improving gastroesophageal mucosal lesions as seen under a gastroscope. Ligan Hewei therapy+PPIs could be a better choice for patients with acid regurgitation and heartburn. These findings could provide physicians and patients with appropriate treatments based on the specific characteristics of GERD. However, additional high-quality RCTs should be conducted to offer more powerful evidence for future research.

## Data Availability Statement

All datasets presented in this study are included in the article/**Supplementary Material**.

## Author Contributions

Conceived and designed the study: LH and LL. Performed the experiment: Y-KD, Y-BW, and HW. Analyzed the data: Y-KD and W-BW. Wrote the paper: Y-KD. Study supervision: RL-L, W-JC, CT, LL, and LH. All authors contributed to the article and approved the submitted version.

## Funding

This work was supported by the National Natural Science Foundation of China (No. 81774238, 81373563 and 30772689), Guangdong Science and Technology Project South China Cooperative Innovation Center for Chinese Medicine (No. 2014B090902002), Construction of Chinese First-Class Discipline Research of Key Project of Guangzhou University of Chinese Medicine ([2020] No. 62, [2019] No. 5 and [2018] No. 6), Construction of Chinese First-Class Discipline of Guangzhou University of Chinese Medicine (2017, No. 70), Construction of High-level University of Guangzhou University of Chinese Medicine (2016, No. 64), and Innovation Team to Foster Scientific Research Projects of Guangzhou University of Chinese Medicine (No. 2016KYTD07).

## Conflict of Interest

The authors declare that the research was conducted in the absence of any commercial or financial relationships that could be construed as a potential conflict of interest.
