# Supplementary material for: Different Traditional Herbal Medicines for the Treatment of Gastroesophageal Reflux Disease in Adults
Source: Front Pharmacol. 2020 Jul 16;11:884. doi: 10.3389/fphar.2020.00884 (PMC7378538; doi:10.3389/fphar.2020.00884)
Supplement: Supplementary file 1 [file DataSheet_1.docx]

**Search strategy for each database**

**PubMed:**

#1 Gastroesophageal reflux disease [SH]

#2 non-erosive reflux disease [SH]

#3 reflux esophagitis [SH]

#4 Barrett esophagus [SH]

#5 exp GERD [SH]

#6 exp NERD [SH]

#7 exp RE [SH]

#8 exp BE [SH]

#9 #1 or #2 or #3 or #4 or #5 or #6 or #7 or #8

#10 traditional Chinese medicine [SH]

#11 exp Chinese herbal medicine [SH]

#12 exp Chinese medicinal herb [SH]

#13 exp traditional Chinese herbal formula [SH]

#14 exp TCM prescriptions [SH]

#15 #10 or #11 or #12 or #13 or #14

#16 randomized controlled trial [PT]

#17 controlled clinical trial [PT]

#18 randomized [TIAB]

#19 #16 or #17 or #18

#20 #9 and #15 and #19

Annotation: Mesh Subheadings [SH] Publication Type [PT] Title/Abstract [TIAB]

**MEDLINE:**

1. exp Gastroesophageal reflux disease.mp.

2. (Gastroesophageal reflux disease OR non-erosive reflux disease OR reflux esophagitis OR Barrett esophagus OR GERD OR NERD OR RE OR BE) .tw.

1. 1 OR 2
2. exp Complementary Therapies.mp.
3. (Therapies, Complementary OR Therapy, Complementary OR Complementary Medicine OR Medicine, Complementary OR Alternative Medicine OR Medicine, Alternative OR Alternative Therapies OR Therapies, Alternative OR Therapy, Alternative).tw.
4. 4 OR 5
5. Randomized controlled trial.pt.
6. controlled clinical trial.pt.
7. randomized.ti,ab.
8. placebo.ti,ab.
9. randomly.ti,ab.
10. trial.ti,ab.
11. groups.ti,ab.
12. OR/7-13
13. 3 AND 6 AND 14

**EMBASE:**

1 (Gastroesophageal reflux disease or non-erosive reflux disease or reflux esophagitis or Barrett esophagus).af.

2 exp GERD/ or exp NERD/ or exp RE/ or exp BE/

3 1 or 2

4 (traditional Chinese medicine).af.

5 exp Chinese herbal medicine/ or exp Chinese medicinal herb/ or exp traditional Chinese herbal formula/ or exp TCM prescriptions/

6 4 or 5

7 (random* OR factorial* OR crossover*).af.

8 exp crossover-procedure/ or exp double-blind procedure/ or exp randomized controlled trial/ or single-blind procedure/

9 7 or 8

10 3 and 6 and 9

**Cochrane Library:**

1. MeSH descriptor: [Gastroesophageal reflux disease] explode all trees
2. (Gastroesophageal reflux disease or non-erosive reflux disease or reflux esophagitis or Barrett esophagus or GERD or NERD or RE or BE):ti,ab,kw (Word variations have been searched)

3. #1 or #2

4. MeSH descriptor: [Complementary Therapies] explode all trees

5. (Therapies, Complementary or Therapy, Complementary or Complementary Medicine or Medicine, Complementary or Alternative Medicine or Medicine, Alternative or Alternative Therapies or Therapies, Alternative or Therapy, Alternative):ti,ab,kw (Word variations have been searched)

6. #4 or #5

7. #3 and #6

**Clarivate:**

1. exp Gastroesophageal reflux disease.mp.

2. (Gastroesophageal reflux disease OR non-erosive reflux disease OR reflux esophagitis OR Barrett esophagus OR GERD OR NERD OR RE OR BE) .tw.

1. 1 OR 2
2. exp Complementary Therapies.mp.
3. (Therapies, Complementary OR Therapy, Complementary OR Complementary Medicine OR Medicine, Complementary OR Alternative Medicine OR Medicine, Alternative OR Alternative Therapies OR Therapies, Alternative OR Therapy, Alternative, Traditional Chinese Medicine OR Chinese Herbal Medicine OR Chinese Medicinal Herb OR Traditional Chinese Herbal Formula OR TCM Prescriptions).tw.
4. 4 OR 5
5. Randomized controlled trial.pt.
6. controlled clinical trial.pt.
7. randomized.ti,ab.
8. placebo.ti,ab.
9. randomly.ti,ab.
10. trial.ti,ab.
11. groups.ti,ab.
12. OR/7-13
13. 3 AND 6 AND 14

**Scopus:**

#1 Traditional Chinese medicine OR Chinese herbal medicine OR Chinese medicinal herb OR traditional Chinese herbal formula OR TCM prescriptions

#2 Gastroesophageal reflux disease OR non-erosive reflux disease OR reflux esophagitis OR Barrett esophagus

#3 randomized controlled trial OR controlled clinical trial OR randomized

#4 #1 AND #2 AND #3

**CNKI** (China National Knowledge Infrastructure):

#1 traditional Chinese medicine

#2 Gastroesophageal reflux disease or non-erosive reflux disease or reflux esophagitis or Barrett esophagus or GERD or NERD or RE or BE

#3 randomized controlled trial OR controlled clinical trial OR randomized

#4 #1 AND #2 AND #3

**Wanfang:**

#1 traditional Chinese medicine

#2 Gastroesophageal reflux disease or non-erosive reflux disease or reflux esophagitis or Barrett esophagus or GERD or NERD or RE or BE

#3 randomized controlled trial OR controlled clinical trial OR randomized

#4 #1 AND #2 AND #3

**VIP** (Chinese Scientific Journals Database):

#1 traditional Chinese medicine

#2 Gastroesophageal reflux disease or non-erosive reflux disease or reflux esophagitis or Barrett esophagus or GERD or NERD or RE or BE

#3 randomized controlled trial OR controlled clinical trial OR randomized

#4 #1 AND #2 AND #3
