# Supplementary material for: Different Traditional Herbal Medicines for the Treatment of Gastroesophageal Reflux Disease in Adults
Source: Front Pharmacol. 2020 Jul 16;11:884. doi: 10.3389/fphar.2020.00884 (PMC7378538; doi:10.3389/fphar.2020.00884)
Supplement: Supplementary file 2 [file DataSheet_2.docx]

**Table A. Overall clinical efficacy.**

| **Side** | **Direct** | | **Indirect** | | **Difference** | | | **tau** |
| --- | --- | --- | --- | --- | --- | --- | --- | --- |
|  | **Coef** | **Std.Err** | **Coef** | **Std.Err** | **Coef** | **Std.Err** | **P>\|z\|** |  |
| **A D*** | 1.126011 | .7357863 | .472349 | 234.8219 | .6536623 | 234.823 | .998 | .1573883 |
| **B F*** | -.7898927 | .4387637 | .5472617 | 1068.056 | -1.337154 | 1068.057 | .999 | .1573874 |
| **C F*** | -1.197703 | .8550628 | .3411563 | 2342.878 | -1.53886 | 2342.878 | .999 | .1573672 |
| **D F*** | -.8146173 | .2772498 | -1.652833 | 351.2056 | .8382154 | 351.2055 | .998 | .1573866 |
| **D G*** | -1.027931 | .379473 | -2.127543 | 907.734 | 1.099612 | 907.7341 | .999 | .1573844 |
| **E F*** | -.8326133 | .5750057 | .5264615 | 1382.238 | -1.359075 | 1382.238 | .999 | .1573874 |

**Annotation:** A: GMD; B: Jianpi-therapy; C: Jianpi-therapy+PPIs; D: Ligan-Hewei-therapy; E: Ligan-Hewei-therapy+PPIs; F: PPIs; G: PPIs+GMD.

**Table B. Efficacy under gastroscope.**

| **Side** | **Direct** | | **Indirect** | | **Difference** | | | **tau** |
| --- | --- | --- | --- | --- | --- | --- | --- | --- |
|  | **Coef** | **Std.Err** | **Coef** | **Std.Err** | **Coef** | **Std.Err** | **P>\|z\|** |  |
| **A D*** | 1.781418 | .6887093 | -.694764 | 1573.907 | 2.476182 | 1573.908 | .999 | 9.49e-09 |
| **B F*** | - | - | - | - | - | - | - | - |
| **C F*** | - | - | - | - | - | - | - | - |
| **D F*** | - | - | - | - | - | - | - | - |
| **D G*** | .1112256 | .6302138 | -.8871711 | 454.1341 | .9983967 | 454.1346 | .998 | 2.23e-07 |
| **E F*** | -1.359638 | .476969 | .4014817 | 1224.622 | -1.761119 | 1224.622 | .999 | 1.55e-07 |

**Annotation:** A: GMD; B: Jianpi-therapy; C: Jianpi-therapy+PPIs; D: Ligan-Hewei-therapy; E: Ligan-Hewei-therapy+PPIs; F: PPIs; G: PPIs+GMD.

**Table C. Improvement of acid regurgitation.**

| **Side** | **Direct** | | **Indirect** | | **Difference** | | | **tau** |
| --- | --- | --- | --- | --- | --- | --- | --- | --- |
|  | **Coef** | **Std.Err** | **Coef** | **Std.Err** | **Coef** | **Std.Err** | **P>\|z\|** |  |
| **A D*** | .9400001 | .677544 | -.2500843 | 49.20836 | 1.190084 | 49.21303 | .981 | .4943608 |
| **B D*** | -.14 | .5030477 | 1.095404 | 86.07486 | -1.235404 | 86.07633 | .989 | .4943417 |
| **B E*** | .189909 | .3601128 | -2.160603 | 179.6765 | 2.350512 | 179.6769 | .990 | .4943392 |
| **C D*** | -.5800002 | .4970151 | 1.870327 | 126.7023 | -2.450328 | 126.7033 | .985 | .4943508 |

**Annotation:** A: Jianpi-therapy; B: Ligan-Hewei-therapy; C: Ligan-Hewei-therapy+PPIs; D: PPIs; G: PPIs+GMD.

**Table D. Improvement of heartburn.**

| **Side** | **Direct** | | **Indirect** | | **Difference** | | | **tau** |
| --- | --- | --- | --- | --- | --- | --- | --- | --- |
|  | **Coef** | **Std.Err** | **Coef** | **Std.Err** | **Coef** | **Std.Err** | **P>\|z\|** |  |
| **A C*** | -.19 | .3131433 | -.3737664 | 89.81416 | .1837664 | 89.8147 | .998 | .2915768 |
| **A D*** | - | - | - | - | - | - | - | - |
| **B C*** | -.7599999 | .3008456 | -.3924049 | 185.7695 | -.367595 | 185.7697 | .998 | .2915745 |

**Annotation:** A: Ligan-Hewei-therapy; B: Ligan-Hewei-therapy+PPIs; C: PPIs; D: PPIs+GMD.
